# A comparative analysis of pharmacists’ perspectives on codeine use and misuse – a three country survey

**DOI:** 10.1186/s13011-018-0149-2

**Published:** 2018-03-27

**Authors:** Tara Carney, John Wells, Charles D. H. Parry, Padraig McGuinness, Richard Harris, Marie Claire Van Hout

**Affiliations:** 1 Alcohol, Tobacco and Other Drug Research Unit, South African Medical Research Council, Francie Van Zijl Drive, Parow, South Africa; 2 Department of Psychiatry and Mental Health, University of Cape Town, Cape Town, South Africa; 3 School of Health Sciences, Waterford Institute of Technology, Waterford, Ireland; 4 Department of Psychiatry, Stellenbosch University, Stellenbosch, South Africa; 5 CARA Pharmacy, Waterford, Ireland; 6 Weldricks Pharmacy, Sheffield, UK; 7 Public Health Policy, Liverpool John Moore’s University, Liverpool, UK

**Keywords:** Codeine, Pharmacy staff, Opinions, Medications

## Abstract

**Background:**

The misuse of codeine is of increasing concern in a number of countries, particularly as this relates to over -the-counter pain and cough relief medication, and is also supplied as a prescription medicine. The study aimed to obtain and analyse the opinions and experience of pharmacy staff with regard to codeine misuse.

**Methods:**

A cross-sectional web-based survey of pharmacy staff’s perspectives on this issue was administered through professional or regulatory bodies and completed by samples drawn in South Africa (*n* = 124), Ireland (*n* = 464) and the United Kingdom (*n* = 129).

**Results:**

The majority of participants reported combination codeine-containing products as most popular, but significantly more pharmacy staff in South Africa reported codeine-containing cough syrups as most commonly popular (X^2^ = 122.7(2), *p* < 0.001). Codeine use was also seen significantly more of a public health problem in South Africa than in the other two countries (X^2^ = 7.6(2), *p* = 0.02). There was no difference across countries in the level of codeine misuse reported by pharmacy staff. Further findings indicate that professional training and education is desired, with unequivocal findings for the need for greater codeine control (X^2^ = 12.0(2), *p* = 0.002).

**Conclusion:**

In conclusion, there were some inter-country differences, but overall the findings seem to suggest that pharmacists across all three countries view codeine misuse as a problem among their customers. Recommendations centre on risk management, surveillance and staff training**.**

## Background

Global public health and medicines regulatory concerns are increasingly focused on the issue of the intentional or unintentional misuse of pharmaceutical opioids [1–3]. Misuse of medicines, including codeine, is defined as ‘the problematic consumption outside of acceptable medical practice or medical guidelines, when self-medicating at higher doses and for longer than is advisable, for intoxicating purposes and when risks and adverse consequences outweigh the benefits’ [1].

Codeine (3-methylmorphine) is one of the most widely available and consumed opiates worldwide, and is most commonly used for its analgesic, antitussive and anti-diarrhoeal properties [4, 5]. Although viewed as a weak opiate, it still has the potential for misuse and abuse or dependence and has a number of associated side effects, such as sedation, euphoria and constipation [6]. Long term or high dose use of combination products containing codeine with ibuprofen or paracetamol can also lead to a number of side effects [6] such as inflammatory bowel conditions, nephrotoxicity, hypokalemia, pancreatitis, gastric ulcers, gastrointestinal haemorrhage, medication over-use, headache, depression and paracetamol hepatotoxicity [7]. Physical tolerance develops over time and unpleasant withdrawal type physical effects can occur when usage stops [6, 8].

While codeine may be prescribed by medical professionals for management of moderate pain or its antitussive properties, it is widely available in some countries in over-the-counter (OTC) preparations, albeit in lower dosages or combination products, including Ireland, South Africa and the United Kingdom (UK). This availability is associated with public lack of awareness of its potential for habit forming use, risk of development of tolerance and dependence with related health harms particularly relating to excessive or long-term use [9–12].

Opiate abuse occurs when codeine-containing products are used for recreational and excessive dose purposes as opposed to medical purposes [4], and can lead to an opioid disorder [13]. Codeine is available to the public over-the-counter and without a doctor’s prescription in a number of countries, which complicates consumer recognition of problem use itself [14–17]. Previous studies have illustrated how individuals with iatrogenic dependence post-medical use for pain, anxiety or sleeplessness, may experience ‘blurring’ between therapeutic and problematic use over time [8, 18].

The regulation of codeine-containing medicines varies by country, with certain countries such as Australia recently restricting availability to prescription-only status following other European countries such as Austria and Italy [19]. Calls for revised scheduling and enhanced surveillance and pharmaco-vigilance have been made in countries where OTC non-prescription misuse of combination analgesics is rising [20, 21]. A recent qualitative study in South Africa, UK and Ireland indicated that pharmacist opinions about up-scheduling OTC codeine are mixed [14]. This is coupled with emerging evidence that codeine misuse is increasingly becoming an issue in these countries, with treatment demand data (the number of people seeking treatment for codeine either as a primary or secondary drug of choice) starting to show an increase in trend in all three jurisdictions [19, 22, 23].

### Aim of the study

The specific aim of this study was to consider codeine misuse and the potential control of and management of codeine in three counties according to pharmacy staff. Specific objectives included investigating pharmacists’ views on codeine use as a significant health problem, misuse and dependence, their level of skills and expertise that can equip them for dealing with presented codeine misuse and dependence, and where future training might be helpful.

## Methods

### Design

The study comprised a cross- sectional survey conducted in the UK, Ireland and South Africa. It was undertaken as part of a large scale multi-country effort (CODEMISUSED Project) to investigate codeine use, misuse and dependence (see Table 1 for regulatory differences between these three countries).

**Table 1:** Codeine Regulations in Ireland, South Africa and the UK

Ireland	Irish medicine board. Guided under the misuse of drugs act 1977.	Over the counter sales permissible in regulated pharmacies under supervision of the pharmacists without prescription Usually in 8 mg/500 and 12.8 mg/500 combination drugs containing analgesics such as paracetamol and aspirin. Cannot be visually displayed or advertised to the consumer. Patient must be advised on its use at point of sale Must contain warning of addiction on pack.	Higher strength codeine formulations 15/300, 30/500 of codeine phosphate/paracetamol combinations- are prescription only medicines. Codeine is also available combined with Ibuprofen; a common formulation is 12.8 mg Codeine alongside 200 mg Ibuprofen. Preparations containing pure codeine (e.g., codeine phosphate tablets is considered controlled drug (CD) (Controlled Drug (Possession without authority is illegal).
South Africa	South African medicines agency.	Over the counter preparations in combination with one or more therapeutically active substances, and containing 20 mg or less of codeine (calculated as base) per dosage unit, . Pack sizes sold can contain up to 100 doses. Sold under supervision of the pharmacists. Sales of codeine containing products must be recorded in the pharmacy.	Prescription Only Medication (POM) medicine in doses up to 20 mg of codeine per dosage unit and are only available on medical prescription.
United Kingdom	Medicine and healthcare products regulatory authority Controlled under the Medicine of Drugs Act 1971.	Over the counter sales permissible in regulated pharmacies under supervision of the pharmacists without prescription. Usually in 8/500 and 12.8/500 combination drugs containing analgesics such as paracetamol and aspirin. 1 over the counter Dihydrocodeine product containing 7.46 mg of active ingredient. Only 1 pack × 32 tablets allowed per customer transaction unless sanctioned by the pharmacist. Can be visually displayed and advertised to the consumer at the point of sale, under pharmacist supervision. Must contain warning of addiction on pack.	Higher strength codeine formulations 15/300, 30/500 of codeine phosphate/paracetamol combinations are prescription only medicines (POM). Codeine is also available combined with Ibuprofen; a common formulation is 12.8 mg Codeine alongside 200 mg Ibuprofen. Preparations containing pure codeine (e.g., codeine phosphate tablets is considered a controlled drug (CD). (Possession without authority is illegal). No longer permitted for use under the age of 18 years (codeine containing linctus for cough).

### Study population

Eligible subjects were either registered pharmacists or pharmacy assistants or technicians as regulated by the appropriate pharmaceutical associations in each country. Retail pharmacy staff not involved in the dispensing or sale of medication were excluded from this study. Participants were identified by professional/regulatory bodies or pharmacy trade organisations in each of the three participating countries: The Pharmaceutical Society of South Africa (PSSA), the Independent Company Chemist Alliance (ICCA) in the UK and the Pharmaceutical Society of Ireland (PSI). The PSSA is an independent professional body that represents pharmacists and pharmacists’ assistants in South Africa. An estimated 7018 individuals are registered in South Africa with the PSSA.The ICCA is an independent body whose members are in the privately owned regional multiple pharmacy sector. It represents over 600 pharmacies throughout the United Kingdom of Great Britain and Northern Ireland. Similarly, the PSI is an independent statutory body that is charged with, and accountable for, the effective regulation of pharmacy services in Ireland. There are 1843 pharmacies in Ireland registered with the PSI, with 5280 registered pharmacists and 452 registered pharmaceutical assistants.

### Web-based survey instrument

A web-based survey was developed, and the preliminary questionnaire was sent out to pharmacy and research staff partners on the CODEMISUSED Project for feedback. The questionnaire was then revised, and sent to the expert advisory panel consisting of experts in pharmacology, addiction and pharmacovigilance. The web-based survey was developed and hosted on a secure website hosted at the South African Medical Research Council, with only the lead author (TC) having direct access to the data.

The survey was then pilot-tested among 15 pharmacists across the three countries, and the only suggestions to modify the questionnaire were minor editing issues. The link to the finalised questionnaire was: http://codemisused.mrc.ac.za which took participants to the homepage. This was put online in March 2015.

The final survey had 24 items with each having various response categories, including ‘yes/no’ responses, options to select all that apply and open-ended questions. The survey was divided into four sections on demographics, codeine misuse as a public health problem and the impact of this, risk management and innovation. Questions included participants’ views on their experiences of codeine misuse, and training for dealing with issues as well as recommendations for innovation.

### Procedures

Ethics approval was provided by the research ethics committees of Waterford Institute of Technology in Ireland; King’s College London in the UK and the South African Medical Research Council in South Africa.

In South Africa, the PSSA included a link to the online survey in their annual newsletter. The The director of PSSA included a link to the survey in the annual newsletter, which all pharmacist and pharmacist’s assistants that are registered with the Society receive, to the online questionnaire for WP4.

In addition, a list of registered pharmacists and pharmacy assistants were emailed the link to the survey and an information letter from an email address especially formulated for this study. In the UK, members of the ICCA were sent an information letter and the link to the online survey. In Ireland the PSI sent out an email to each registered pharmacist and pharmaceutical assistant requesting them to complete the survey, as well as the information letter and the link to the online questionnaire.Participants were asked to read an information sheet which provided further details about the aims of the study. Before participants could progress to participating in the actual survey they had to tick each box to say that they agreed that they understand they had read the information sheet, their participation was voluntary, they understood the study and that their identity would be kept confidential. Informed consent was therefore obtained from all individual participants included in the study.

### Data analysis

Quantitative data from the survey was first analysed using descriptive statistics using SPSS version 23. The data was then analysed to compare any country differences. Since the quantitative data was non-normally distributed, any continuous data was compared by using the Kruskal-Wallis H statistical test. *P*-values of pairwise categorical (yes/no-type) count data were calculated using Pearson Chi-square test statistics with the exception of the amount of codeine misused which was ordinal data (low, medium, high). In this case, the Linear-by-Linear Association test statistics were used.

## Results

There were 464 Irish participants, 123 participants from South Africa and 129 participants from the UK. Table 2 shows the personal demographics of the participants and types of pharmacies in which they work. Across sites, the majority of participants were female and aged between 20 and 39 years old. There were no significant differences in the gender breakdown between countries (*X*^2^ = 2.54, df = 2, *p* = 0.28), but there were significant age differences (*X*^2^ = 27.81, df = 2, *p* < 0.001). South African participants reporting a significantly higher average number of years in practice than those in Ireland and the UK (*X*^2^ = 42.03, df = 2, *p* < 0.001).

**Table 2:** Demographics of pharmacy staff across the three countries and cross-country comparisons

Demographic Characteristics	Ireland (*n* = 464) (*n*,%)	South Africa (*n* = 123) (*n*,%)	UK (*n* = 129) (*n*, %)	Test statistic χ2(*df*)	*p*-value
Gender					
Male	168 (36.2%)	53 (43.1%)	44 (34.1%)		0.28
Female	296 (63.8%)	70 (56.9%)	85 (65.9%)	2.54 (2)	
Age					
20–39	278 (59.9%)	41 (33.3%)	72 (55.8%)	41.70 (2)	< 0.001*
40+	186 (40.1%)	82 (66.7%)	57 (44.2%)		
Number of years in practice: Median (Range)	12 (1–49)	25 (0–49)	15 (1–48)	42.03 (2)	< 0.001*
Type of location					
Urban	261 (56.3%)	84 (68.3%)	75 (58.6%)	11.03 (4)	0.02*
Rural	99 (21.3%)	17 (13.8%)	17 (13.3%)		
Both	102 (22.0%)	21 (17.1%)	36 (28.1%)		
Missing	2	1	1		
Type of pharmacy					
Community/retail	400 (86.2%)	98 (83.1%)	117 (95.1%)	8.80 (2)	0.01*
Others (Academic, Hospital, More than one type of pharmacy)	61 (13.1%)	24 (16.9%)	10 (4.9)		
Missing	3	1	2		
Position					
Full-time	317 (68.3%)	87 (71.9%)	91 (70.5%)	21.65 (6)	< 0.001*
Part-time	57 (12.3%)	4 (3.3%)	16 (12.4%)		
Locum	67 (14.4%)	14 (11.6%)	13 (10.1%)		
Pharmacy assistant	20 (4.3%)	16 (13.2%)	9 (7.0%)		
Nearby pharmacies					
0	49 (10.7%)	17 (13.8%)	12 (9.3%)	53.54 (8)	
1–5	175 (38.0%)	76 (61.8%)	82 (63.6%)		< 0.001*
6–10	140 (30.4%)	26 (21.1%)	19 (14.7%)		
11–19	61 (13.3%)	3 (2.4%)	11 (8.5%)		
20 or more	35 (7.6%)	1 (0.8%)	5 (3.9%)		
	448 (98.0%)	120 (99.2%)	125 (98.4%)	0.76 (2)	0.68

*p-value is significant

While the majority of participants reported working in urban settings, with significant differences noted between sites (*X*^2^ = 11.03, df = 4, *p* = 0.02). Most were full-time pharmacists in community/retail pharmacies, with a small number working in other settings such as academic or hospital settings, and significant differences between the three countries (*X*^2^ = 8.80, df = 2, *p* = 0.01).

There were also some significant country differences between the number of pharmacies close to (within a 3 km radius) the participants’ workplace, (*X*^2^ = 53.54, df = 6, *p* < 0.001), with seemingly few surrounding pharmacies in South Africa.

While a small proportion of participants in Ireland (*n* = 8, 1.8%) and the UK (*n* = 1, 0.8%) reported that cough and cold syrups containing codeine are popular products sold in their pharmacy, this proportion was significanlty larger in South Africa (*n* = 34, 27.9%) (Table 3). In all three countries combination medication (that is, those containing codeine together with other ingredients such as paracetamol and ibuprofen) were mentioned as being most popular (Ireland: *n* = 448, 98.2%; South Africa: *n* = 88, 72.1%; UK: *n* = 128, 99.2%), although this was significantly lower in South Africa (*X*^2^ = 122.7(2), *p* < 0.001) (see Table 3).

**Table 3:** Pharmacy perspectives of codeine as a popular product or problem and cross-country comparisons

	Ireland (*n* = 464) (*n*, %)	South Africa (*n* = 123) (*n*, %)	UK (*n* = 129) (*n*, %)	Test statistic χ2(df)	*p*-value
Most popular product					
Cough/cold syrup	8 (1.8%)	34 (27.9%)	1 (0.8%)	122.7 (2)	< 0.001*
Combination medication	448 (98.2%)	88 (72.1%)	128 (99.2%)		
Missing	10	1	0		
Codeine as significant health problem	302 (65.1%)	91 (74.0%)	75 (58.1%)	7.6 (2)	0.02*
Amount of codeine misused					
Low	177 (38.2%)	40 (32.5%)	59 (45.7%)		
Medium	204 (44.9%)	56 (46.3%)	59 (45.7%)		
High	73 (16.1%)	25 (20.7%)	10 (7.8%)		
Missing	12	2	1	2.9	0.09

*p-value is signficant

**Fig. 1:**
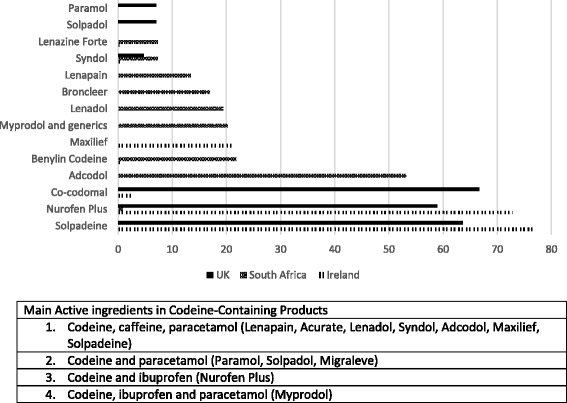
Most commonly used codeine-containing products reported by pharmacy staff across three countries

The trade names of popular products containing codeine differed between the three countries. The majority of Irish participants reported that Solpadeine® (*n* = 355, 76.7%) and Nurofen Plus® (*n* = 337, 72.8%) were the most popular codeine-containing OTC products sold, followed by Maxilief® (*n* = 97, 21.0%). In South Africa, participants mentioned a number of popular products including Adcodol® (*n* = 66, 53.2%) and Lenadol® (*n* = 24, 19.4%) and also mentioned cough and cold syrups such as Benylin with Codeine® (*n* = 27, 21.8%) and Broncleer with Codeine® (*n* = 21, 16.9%). In the UK, Co-codamol (*n* = 86, 66.7%), Solpadeine® (*n* = 82, 63.6%) and Nurofen Plus® (*n* = 76, 58.9%) were the most popular products (see Fig. 1).

The majority of participants saw codeine misuse as a public health issue, with the highest proportion of participants in South Africa reporting codeine misuse as a problem (*n* = 91, 74.0%). Almost two-thirds of participants in Ireland (*n* = 302, 65.1%) and just over half reporting this in the UK (*n* = 75, 58.1%) viewed it as a public health issue. These proportions were significantly different across the three countries (*X*^2^ = 7.6 (2), *p* = 0.02).

Respondents across all three countries reported that a low to medium amount of codeine sold in their pharmacies is misused, with no significant differences across South Africa, Ireland and the United Kingdom.

The majority of participants reported that they had not received any kind of specialist training in substance use issues (Table 4). In Ireland, only 69 (15.0%) had received training, which was significantly lower than in South Africa (*n* = 31, 25.2%) and the UK (*n* = 32, 24.8%) (*X*^2^ = 10.9(2), *p* = 0.004). Most of the participants reported a further need for training and education (Ireland: *n* = 284, 61.5%, South Africa: *n* = 87, 71.3%, UK: *n* = 81, 62.8%) with no country differences (*X*^2^ = 4.1(2), *p* = 0.13).

**Table 4:** Existing resources for pharmacy staff and cross-country differences

	Ireland (*n* = 464) (*n*, %)	South Africa (*n* = 123) (*n*, %)	UK (*n* = 129) (*n*, %)	Test statistic χ^2^(df)	p
Specialist addiction training	69 (15.0%)	31 (25.2%)	32 (24.8%)	10.9 (2)	0.004*
Need for training and education	284 (61.5%)	87 (71.3%)	81 (62.8%)	4.1 (2)	0.13
Need for public education	393 (85.4%)	113 (91.1%)	100 (78.1%)	8.5 (2)	0.01*
Existing risk management system	177 (39.0%)	60 (50.4%)	66 (51.6%)	9.5 (2)	0.009*
Willingness to participate in centralized monitoring system	333 (74.5%)	96 (80.0%)	107 (83.6%)	5.3 (2)	0.07
Belief that current level of codeine control in jurisdiction is high enough	192 (42.0%)	50 (41.0%)	75 (58.6%)	12.0 (2)	0.002*

*p-value is signficant

Participants were also asked if education and support materials on substance use would be useful for pharmacy customers and the general public (Table 4). There were significant inter-country differences, with the highest proportion expressing need for such public education in South Africa (*n* = 113, 91.1%), followed by Ireland (*n* = 393, 85.4%), and then the UK (*n* = 100, 78.1%) (*X*^2^ = 8.5(2), *p* = 0.01).

When asked about existing risk management systems (that flags customers who may be abusing codeine, 51.6% of participants in the UK (*n* = 66) reported that this system exists in their country. A similar proportion (*n* = 60, 50.4%) in South Africa reported having a risk management system, while in Ireland this proportion was significantly lower (*n* = 177, 39.0%) (*X*^2^ = 9.5(2), *p* = 0.009). The majority of participants were willing to participate in a centralised system that monitors the provision of codeine products within pharmacies (Ireland: *n* = 334, 71.9%; South Africa: *n* = 95, 77.2%; UK: *n* = 107, 83.0%). There were no significant country differences (*X*^2^ = 5.3(2), *p* = 0.07).

Finally, significantly more pharmacy staff in the UK (*n* = 75, 58.1%) than in Ireland (*n* = 191, 41.2%) or South Africa (*n* = 51, *n* = 41.5%) reported that they believe that the current level of codeine control in their jurisdiction is high enough (*X*^2^ = 12.0(2), *p* = 0.002).

## Discussion

Most pharmacy studies investigating the misuse of pharmaceutical opioids have focused on stronger opioids as opposed to weaker opioids like codeine which are frequently sold over the counter as combination pharmaceuticals. This study is one of the first in these countries conducted with pharmacy staff solely on the topic of codeine use and misuse, as well as the different countries’ response to codeine misuse. The findings indicate that the misuse of codeine is seen as a significant public health issue by pharmacy staff, and also indicated a need for training in the field of substance use in general.

The majority of participants reported that from their experience, a medium to high amount of codeine provided at pharmacies is misused by clients in all three countries. Concerns from pharmacists around the misuse of codeine-containing products in general by their profession has also been identified in other studies conducted with pharmacists about the abuse of medication [9, 15, 17, 21, 24–27] with the results of a recent Scottish study finding that pharmacists reported codeine-containing products to be the frequently abused form of OTC medication [28]. Coupled with this, participants admitted to gaps in their training on how to identify and address codeine misuse and expressed a willingness to be trained, particularly in monitoring systems. However, findings around the control of codeine were equivocal.

A number of significant inter-country differences were noted, indicating perhaps different methods of dealing with misuse. For example, while all three countries discussed the popularity of codeine-containing combination products, only South African pharmacy staff reported that codeine-containing syrups were popular. There is recent evidence that codeine-containing syrups are increasingly popular with young people especially in relation to youth culture [29]. Elsewhere user preferences have been for codeine-containing tablets [2, 29]. This is partly because cough syrup that contains codeine is less available in the UK and Ireland in comparison to South Africa [29].

Another inter-country difference noted was the proportion of participants who reported that their country had an existing risk management system in place in pharmacies that could be used to limit drug-related harms. In this regard, the highest proportion of participants in the UK reported a willingness to participate in such a monitoring system.

The use of real-time reporting to monitor medications including opiates exists in various countries including the USA, Canada and Australia [2, 30] and has also been recommended based on the findings of a recent scoping review of codeine [31]. This review suggested that prescription drug monitoring through an online system for prescriptions, combined with real time systems for both OTC and prescription codeine-containing products should be developed which could be used in pharmacies. It is suggested that such systems may be useful to track and monitor levels of dispensing and reduce inappropriate prescribing. It is also recommended that they could be used to reduce ‘user shopping’ at multiple prescribers and pharmacies and thereby aid to reduce adverse events such as overdose [32]. This is possibly an innovative approach to dealing with codeine misuse.

The use of real-time monitoring systems can help to ensure that patients who present with genuine therapeutic need receive OTC codeine-based products. This type of system can also assist in identifying possible misuse by flagging consumers that purchase large quantities of codeine-based products. This has been effective for other products such as pseudoephedrine in Australia, where the Project STOP programme is implemented [32], which assists pharmacist in making decisions to dispense of products containing pseudoephedrine or not. In South Africa, such a surveillance system was developed, known as the Codeine Care Initiative [33], which consists of tagging codeine-containing medication in combination with a secure central database to monitor these purchases across the country. However, it has been put on hold. Australia implemented a similar voluntary real-time monitoring program for OTC codeine (MedsASSIST) briefly prior to the decision to up schedule all codeine-containing products to prescription only [34].

Since tracking customers that purchase OTC products containing codeine can be challenging, the study findings also support the consideration of alternative approaches to reduce misuse and dependence on OTC codeine-containing products. These focus on comprehensive care practices within pharmacies including detecting and assessing consumer codeine misuse, the provision of information around risk of dependence and associated harms to clients, and how to prevent adverse effects [3].

Another finding from the current study is that participants reported that pharmacy staff lacked training, but wanted specific training around substance use issues, including codeine. Previous studies have recommended that increased practice emphasis on specific pharmacist and counter staff training in addiction, mental health and communication or conflict resolution skills should be considered [9, 20, 24, 35–37]. In addition, pseudo-patron visits [37] as a training tool in adherence to standards of practice can provide immediate feedback, and result in positive changes in quality of service in pharmacy practice [38, 39].

### Limitations

One of the limitations of the current study was the low response rate in both South Africa and the UK. In Ireland, the authors had established a relationship with a key staff member at the PSI, which led to the survey being sent out by the professional body first hand, and also allowed for intermittent reminders to be sent out to complete the survey. The lower response rate was, however, not from the lack of trying on the authors’ part in the other two countries. In the UK, the General Pharmaceutical Council only provides physical addresses of pharmacies for research purposes. The South African Pharmacy Council was willing to put a link in their newsletter that went out, but not send the questionnaire to registered pharmacy staff. It must be noted that paper questionnaires could have been posted to the pharmacies in the UK and Ireland, but since this was a web-based survey it would not have been advisable to use a mixed-methods approach. As mentioned earlier, one of the limitations was the low response rate in South Africa and the UK. It is possible that future studies be conducted that are only paper-based to ascertain whether this will lead to a larger sample size, and greater generalisation.

Due to this small size and since the study was only conducted in three countries, we acknowledge that it is not possible to generalise the current findings to the general pharmacy community in a) these three countries or b) other countries beyond the three included in the sample. Despite this, the study still offers key insights into the comparable experiences of pharmacy in dealing with the public health issue of codeine misuse.

## Conclusion

Misuse of opioid analgesics is an emergent global public health concern. This study represents the first attempt to compare pharmacist staff perceptions and experiences across three distinct regulatory regimes providing OTC and prescription codeine. Similar to previous studies in Australia and the UK [17, 20], the response to patient misuse of codeine is a challenge for community pharmacies. Training needs warrant consideration and development in order for pharmacy staff to educate patients, and intervene and support those experiencing problematic codeine use. Risk management and surveillance systems equally warrant development and buy in from regulatory bodies and the retail sector, and should be evaluated as suggested by Shard et al. [40].
